# Design requirements for a digital storytelling application for people with mild cognitive impairment (MCI)

**DOI:** 10.1177/20552076241282237

**Published:** 2024-09-19

**Authors:** Di Zhu, Abdullah Al Mahmud, Wei Liu

**Affiliations:** 1 3783 Centre for Design Innovation, Swinburne University of Technology, Hawthorn, Australia; 2 47836 Beijing Key Laboratory of Applied Experimental Psychology, National Demonstration Centre for Experimental Psychology Education, Beijing Normal University, Beijing, China

**Keywords:** Digital storytelling application, people with MCI, design requirements, qualitative study

## Abstract

**Background:**

The current digital storytelling applications present advantages for individuals with Mild Cognitive Impairment (MCI); however, there exists a notable oversight regarding their potential to facilitate group-based storytelling activities with this population. This study endeavors to identify design requirements for a more inclusive and accessible digital storytelling tool for people with MCI.

**Method:**

The methodological framework encompasses distinct stages, commencing with focus groups and interviews (Stage 1), followed by prototyping workshops (Stage 2) and qualitative prototype testing (Stage 3). The comprehensive three-stage research involved participants residing in Beijing, China, including 43 people with MCI aged 65–95 years (*M* = 79.09, *SD* = 8.99), with a mean Montreal Cognitive Assessment score of 21.91 (range = 18–26, *SD* = 2.40). Additionally, 17 care partners and 10 occupational or clinical therapists actively participated.

**Result:**

The culmination of the three-stage research process has yielded 12 discernible key design requirements. Preferred storytelling themes center around narratives designed to elicit positive emotions. The narrative material generation process involves a systematic approach, unlocking memories through carefully formulated questions. In memory retrieval, users are provided with hints, bolstering confidence and perpetuating a semblance of face-to-face interaction. The focus in story sharing lies in transcending mere narration and extending it to a wider audience.

**Conclusion:**

This case study centers on crafting a digital storytelling application to enhance social connections for people with MCI. It delves into crucial design requirements addressing memory challenges, emphasizing individual preparation and group sharing. The developed digital storytelling application demonstrates potential to offer valuable memory support and foster personal and collective connections. Future research will focus on formal testing to evaluate these outcomes.

## Introduction

### Background

Mild Cognitive Impairment (MCI) constitutes an intermediate state between typical cognitive aging and more pronounced cognitive decline, exemplified by conditions such as dementia.^1^ People with MCI encounter challenges in maintaining social connections and actively participating within communities, a predicament exacerbated by impediments such as social settings. Whether the social settings are familiar or unfamiliar, individuals with MCI often face numerous difficulties, including anxiety, confusion, and difficulty remembering social norms and cues.^2^ Memory deficits and diminished self-efficacy and confidence further hinder their involvement in normal social activities;^3^ diminished self-efficacy, and confidence,^4^ thereby hindering their involvement in normal social activities. These impediments not only detrimentally affect the overall well-being of people with MCI but also pose an elevated risk for exacerbated cognitive decline and heightened social isolation.

Research suggests that socially isolated people with MCI experience reduced psychological well-being and heightened negative emotional states relative to cognitively healthy counterparts in the older adults demographic.^5^ A comprehensive approach involving multifaceted interventions has been identified as conducive to enhancing the quality of life and prolonging independence among those with MCI. In recent years, digital interventions such as digital storytelling^6,7^ and digital reminiscing^8^ have emerged as promising tools to support people with MCI, facilitating the recollection of past experiences, events, and emotions. The interventions employ digital prompts such as photographs, household items, music, and audio recordings.^9^ The contemporary practice involves the digitization of such prompts, rendering them readily accessible.^9^ Among the diverse array of digital platforms, smartphones emerge as the most predominant conduit for digital memory retrieval,^10–12^ closely followed by tablets, which are also cited frequently.^13,14^ Additional modalities enlisted to bolster participation encompass TV,^15^ laptops,^16^ and computers,^17^ with the potential for their synergistic utilization.^11^ Within this context, the active involvement of people with MCI in the design process is critically important and profoundly influences the pertinence and accessibility of storytelling applications. Such technological interventions may uniquely contribute to bridging gaps in social engagement, but also stimulate cognitive abilities and impart a sense of empowerment to those affected by MCI. Furthermore, the advent of social media presents opportunities for people with MCI to share their experiences, thoughts, and feelings with a broader audience, transcending geographical constraints.^18^ Such technological interventions uniquely contribute to bridging gaps in social engagement, stimulating cognitive abilities, and imbuing a sense of empowerment among those affected by MCI.

Technology-based storytelling therapy has demonstrated a notable impact on the affective states and subjective well-being of participants, fostering heightened interpersonal connections with friends and family.^16^ Although prevailing programs typically incorporate preliminary training sessions, they predominantly leverage commercially available technology, presupposing a requisite level of digital proficiency for adept multimedia editing and storytelling.^19^ In certain instances, trained volunteers or care partners are engaged to assist individuals with dementia in their storytelling endeavors.^10^ Nevertheless, the customization of software tailored to the specific needs of people with MCI to enhance usability and mitigate the learning curve represents an area necessitating further investigation.^20^ Evidence suggests that incorporating group-oriented elements in digital storytelling not only enhances engagement but also promotes a sense of community and collective creativity.^21^ Technology enables real-time collaboration, allowing group members to contribute, edit, and enhance narratives collectively.^22^ For the cultivation of social engagement, it is imperative that individuals in close proximity to those with dementia initiate and sustain dialogues.^23^ The preservation of well-being and quality of life for both individuals with cognitive impairment and their care partners hinges upon fostering positive interactions.^24^ Participants in several studies reported strengthened familial and social bonds,^25^ exemplified by the cultivation of intergenerational relationships and heightened interactions with familiar locales, such as community centers, local parks, and familiar neighborhood settings. This innovative form of location-based asynchronous communication serves as a means to effectively enhance familial connections.^16^ Multimedia elements, such as images and videos, can be seamlessly integrated, enriching the storytelling process.^26^ Overall, the integration of technology enhances the accessibility, inclusivity, and creativity of group-based storytelling activities, offering novel avenues for collective expression and shared narratives. However, the current landscape of digital storytelling applications reveals a notable gap in adequately supporting group activities. Existing applications primarily focus on individual interactions,^12,27^ neglecting the potential of technology for supporting collaborative storytelling experiences. Therefore, addressing this gap is essential for creating more inclusive and socially impactful digital storytelling applications.

In summary, while technology holds immense potential to support group-based storytelling activities, a notable gap exists in the current landscape. The predominant focus of existing storytelling applications on commercial purposes underscores the need to specifically address the requirements of people with MCI in group-based storytelling. The current applications often lack features conducive to group interactions and may require additional training or support. Recognizing this gap emphasizes the urgency of developing intuitive, user-friendly applications that cater to the distinct needs of people with MCI, ensuring their meaningful engagement in the digital storytelling realm. By enhancing community-based social participation and cognitive function, the application seeks to provide a tool that fosters social connections and cognitive engagement among people with MCI. This study aims to explore the specific needs of people with MCI when using a digital storytelling application. It also seeks to gather specific suggestions from people with MCI for improving the current interface design of the digital storytelling application.

### Theoretical framework

According to the World Health Organization (WHO), participation is defined as involvement in a life situation. Participation is classified in the International Classification of Functioning, Disability and Health (ICF) within the following domains: learning and application of knowledge; general tasks and demands; communication; mobility; self-care; domestic life; interpersonal interactions and relationships; major life areas (such as work or school); and community, social, and civic life.^28^ Functioning is a complex process that necessitates the acquisition of cognitive, physical, and psychological abilities, as well as the establishment of favorable contextual conditions. The ICF is useful in that it provides a common vocabulary to describe how people live with a health condition.^29^

In the context of enhancing social participation and connection for people with MCI, understanding and applying relevant theoretical models is crucial. The ICF model provides a comprehensive framework for understanding how cognitive impairments impact daily activities and participation in social life. This model helps identify the specific challenges that people with MCI face in their social interactions and day-to-day functioning.

To address these challenges, Social Cognitive Theory (SCT) offers valuable insights. SCT posits that self-efficacy and goals directly influence behavior, while outcome expectancies and socio-structural factors have indirect effects. Goals guide behavior by determining the effort individuals are willing to invest.^30^ Perceived self-efficacy relates to one's belief in their ability to manage tasks.^31^ By focusing on self-efficacy, SCT emphasizes the importance of the individuals’ confidence in their abilities to achieve desired outcomes, which is essential for fostering active participation and engagement.

Huber et al. identified three elements of social health: the capacity to fulfill potential and commitments, competency to manage life independently, and engagement in social activities.^32^ Integrating SCT with the ICF model allows for a comprehensive approach to enhancing social participation. The ICF model outlines the impacts of cognitive impairments, while SCT provides strategies to improve self-efficacy and set social goals, which are crucial for overcoming participation restrictions.

In this study, we combine the ICF and SCT to enhance social participation for people with MCI. The ICF model outlines cognitive impairment impacts, while SCT provides strategies to improve self-efficacy and set social goals. Our technology-based intervention aims to enhance social participation by addressing ICF limitations and boosting self-efficacy. To ensure the effectiveness of such technology, it needs to be co-designed with the end-users. Involving people with MCI in the co-design process ensures that the technology is tailored to their specific needs and preferences, ultimately supporting social participation more effectively. Co-designed technology can better address the nuances of user requirements, leading to higher engagement and satisfaction among end-users.

### Co-design framework

The necessity of the study setup for designing a digital storytelling application for people with MCI is grounded in the principles of Participatory Design (PD).^33^ These approaches ensure that the application is tailored to the specific needs, preferences, and abilities of the target users, thereby enhancing usability, accessibility, and overall user satisfaction. PD involves the active involvement of users and other stakeholders in the design process.^2^ This approach recognizes users as experts in their own experiences and leverages their insights to co-create solutions that are more relevant and effective. Engaging people with MCI and care partners in the design process is essential for understanding the complex and nuanced needs of the target user group.^33^ Their firsthand experiences and insights are invaluable in identifying potential barriers and opportunities for enhancing the usability and effectiveness of the digital storytelling application. The study includes co-design workshops where people with MCI, care partners, and therapists collaborate with researchers to brainstorm ideas, create prototypes, and provide feedback. This participatory approach ensures that the application is designed with a deep understanding of the users’ context, preferences, and challenges, resulting in a more personalized and impactful solution.

By integrating PD approaches, this study aims to create a digital storytelling application that is not only functional and accessible but also resonates with the users’ personal and social needs. The necessity of this study setup lies in its ability to enhance usability, increase engagement, and ensure relevance. Through iterative testing and refinement based on user feedback, the application is continuously improved to ensure it is easy to use and navigate for people with MCI. By involving users in the design process, the application is more likely to include features and functionalities that genuinely engage and support the users in their storytelling activities. The participatory approach ensures that the application addresses real-world challenges and opportunities identified by the users themselves, leading to a more relevant and effective solution. In conclusion, the necessity of the study setup is firmly rooted in the principles of PD, ensuring that the digital storytelling application for people with MCI is designed with a deep understanding of their needs and preferences, ultimately enhancing their cognitive and social well-being.

Co-design elevates the relevance and efficacy of the developed applications. Through direct engagement with people with MCI in the scoping stage, participatory design workshops, prototype development, and usability evaluation,^34,35^ the process captures firsthand insights into their needs, preferences, and challenges. This in-depth understanding proves pivotal in crafting solutions that not only boast technological robustness but also resonate with the authentic experiences of the target users.

Co-design denotes the collaborative engagement of individuals, encompassing users and stakeholders, in the design process. The incorporation of users in design sessions serves to enhance engineers’ and researchers’ understanding of their specific needs. Notably, there exists a proclivity among designers to prioritize safety considerations over refining the product experience for the older adults’ demographic.^36^ While co-creation may ostensibly appear antithetical to speculative design, which involves imagining and exploring future possibilities without the constraints of current technological limitations, both methodologies retain foundational significance in effective design practices. Speculative design is a design approach that focuses on envisioning and creating scenarios for potential futures.^37^ It challenges current assumptions and explores “what if” questions to open discussions about the implications of emerging technologies, societal changes, and innovative ideas. This method encourages designers and stakeholders to think beyond the present and consider long-term impacts and possibilities, fostering creativity and innovation. Given that our proposed solution targets an aging population, a holistic approach necessitates the amalgamation of foresight, insight, and engagement.^35^

Within caregiving processes, considerable attention is directed towards the involvement of individuals with dementia and their families in tailoring care packages, processes and tools. This inclusive approach is designed to establish parity, respect, and hospitality, thereby facilitating a comprehensive analysis of usage patterns, design considerations, and levels of participation.^38^

During the co-design process, the facilitative role assumed by creators marks a departure from the historically ascribed function of a translator, traditionally tasked with translating conceptual ideation to tangible products.^33^ This transformative shift delineates a modification in the designer's perspective from a third-person vantage point to a first-person stance.^39^ The pervasive adoption of co-design methodologies, particularly prevalent in healthcare contexts, facilitates multidisciplinary collaboration, particularly in sensitive or intricate settings.^40^ This approach proves conducive to establishing collaborative partnerships with people affected by MCI, care partners, and family members collectively engaging in the exploration of design solutions.^41^

Vulnerable groups, such as those affected by MCI, undeniably contribute creatively, thereby heightening the significance of equitable collaboration, effective communication, and a shared sense of ownership during co-design sessions.^33^ Notably, research undertaken within the co-design process holds the potential to unveil insights and concepts that surpass the discernible scope of purely verbal interviews, a salient consideration given the potential difficulties faced by people with MCI in recollecting experiences and articulating emotions verbally.^42^ Consequently, designers are empowered to discern needs, intentions, attitudes, abilities and desires, collecting valuable feedback to propose refined design solutions.^36^

The active involvement of people with MCI in the co-design process is not only feasible but also invaluable, contributing directly to technological enhancements and fostering increased adoption of technology among this demographic.^39^ The co-design serves as a conduit for a more nuanced understanding of potential challenges that may impede the optimal storytelling experience. Robinson advocates for a co-design methodology to comprehensively explore the needs of individuals with dementia and their care partners concerning the utilization of assistive technologies.^34^

The active involvement of people with MCI in co-design sessions serves as a means for designers to comprehend the intricate requirements of the user experience, ensuring continuity from the interview phase to the final evaluation. This study seeks to facilitate the participation of those with MCI in co-design sessions, providing a platform for the expression of their feelings and ideas. The adopted co-design methodology encompasses three principal stages: a scoping stage, participatory design workshops, and prototype development. Additionally, a postworkshop stage is introduced for formative qualitative evaluation following prototype development.^35^

Consequently, this study aims to address the following research question: What are the design requirements for a digital storytelling application, as discerned, derived from the aggregation of user feedback and prototype testing?

## Materials and method

### Participants

We employed purposive sampling, a nonprobability sampling method that involves intentionally selecting participants based on specific characteristics or qualities relevant to the research, rather than randomly choosing them from the population. Jingshilaonian, a psychosocial support institution in the Tiantongyuan Community, Beijing, has been enthusiastic about participating in our research. This social work organization advertises project participation through face-to-face meetings and WeChat groups, providing mental health services and daily activity support for older adults. They helped us recruit and screen people with MCI for our study. Inclusion criteria for people with MCI mandated a clinical diagnosis, age 65 or older, residence in Beijing's urban area, absence of visual or hearing disabilities, and proficient reading abilities, especially with digital interfaces. Participants required a willing care partner for the focus group, with exclusions for those with significant neurological conditions (e.g., strokes or brain injuries) that could confound the analysis. Care partners, whether informal (typically relatives) or formal (mainly nurses), were included based on 1 year or more of informal caregiving experience or 3 years or more of formal caregiving experience, encompassing roles of supervision, support, and assistance to people with MCI. Therapists were eligible if they held a master's degree or above, possessed 3 or more years of experience working with people with MCI, and were either occupational or clinical practitioners.

As Table 1 shows, the study involved people with MCI (*n *= 43) aged 65–95 years (*M* = 79.09, *SD* = 8.99), with a mean Montreal Cognitive Assessment (MoCA) score of 21.91 (range = 18–25; *SD* = 2.40). The study also included care partners (*n* = 17) and therapists (*n* = 10) in Beijing, China. Therapists included occupational therapists and clinical psychologists. These professionals were chosen for their expertise in cognitive and functional rehabilitation, which is crucial for developing and evaluating the digital storytelling application. In Stage 1, 31 people with MCI, along with 13 care partners and 10 therapists, participated. Moving to Stage 2, 12 people with MCI and four care partners took part. In Stage 3, the same people with MCI attended the testing session alongside half of the therapists (*n* = 5) from Stage 1. Our research classified digital literacy, which encompasses support for those who need assistance with their devices, proficient users who easily navigate mobile technology, and individuals who do not own a mobile device. Participants in our study were persons with MCI. It is important to clarify that the categories of digital literacy—those who need assistance with their devices, proficient users, and individuals who do not own a mobile device—are mutually exclusive. Each participant reported their digital literacy status by selecting one of the three options listed in Table 1. Results showed that 34.88% of participants did not own a mobile device, 39.54% required support, and 25.58% were proficient users.

**Table 1.** Demographic characteristics of people with MCI.

Characteristic	Group	*n*	%
Gender	Male	16	37.21
	Female	27	62.79
Age (average = 79.09, SD = 8.99)	65–69	6	13.95
	70–74	11	25.58
	75–79	6	13.95
	80–84	7	16.28
	85–90	7	16.28
	Above 90	6	13.95
Digital literacy	Nonowners	15	34.88
	Need support	17	39.54
	Proficient users	11	25.58

*Note*. Non-owners: Participants who do not own any digital devices. Need support: Participants who own digital devices but require assistance in using them. Proficient users: Participants who own and can independently use digital devices without assistance.

This research has received ethical approval from The Swinburne University of Technology's Human Research and Ethics Committee (reference number: 20226525-10539, approval date: 6 July 2022; reference number: 20226525-11105; approval date: 26 September 2022). Additionally, written consent with research goals from all participants has been obtained, ensuring compliance with ethical standards throughout the research process.

### Procedure

**Figure 1.**
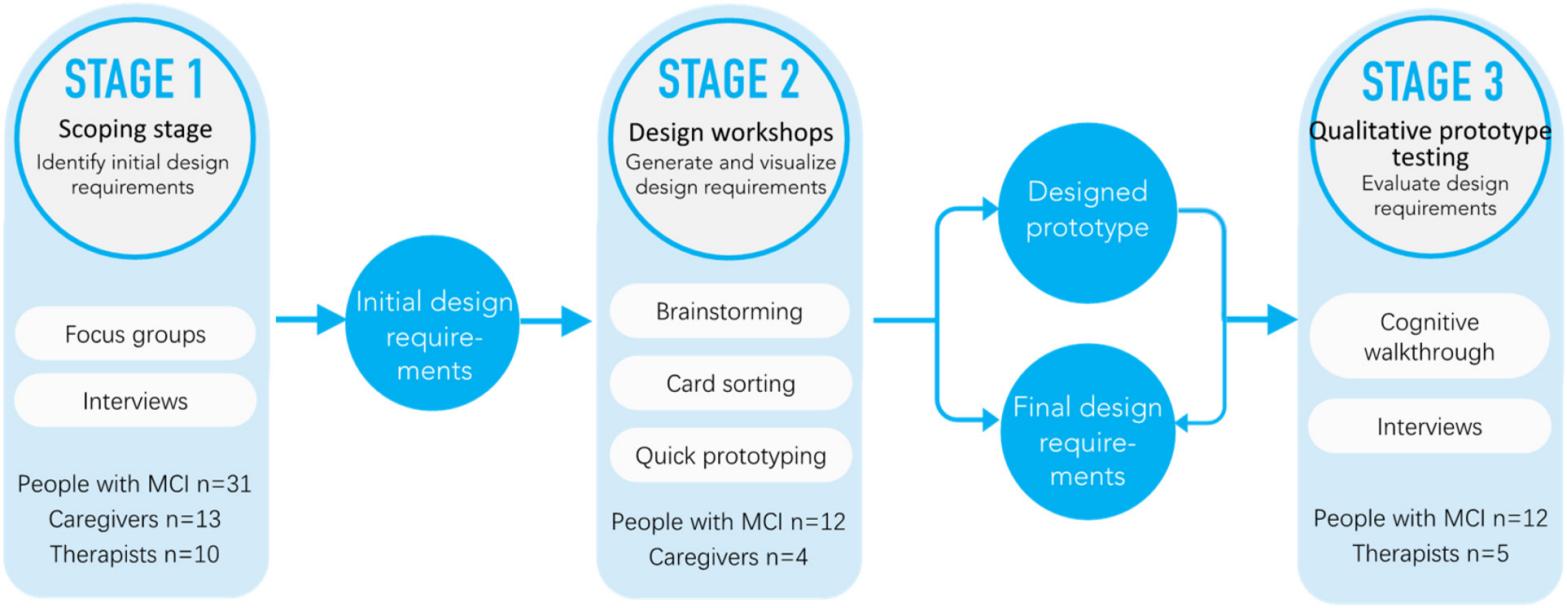
Overview of the research process.

Figure 1 illustrates that the study unfolds in three sequential stages: the scoping stage involving focus groups and interviews (Stage 1), design workshops incorporating activities such as voting, card sorting, and prototyping workshops (Stage 2), and qualitative prototype testing encompassing cognitive walkthroughs and interviews (Stage 3). Each of the three stages was executed on different days. Details of stages could be referred to the protocol paper.^43^ Before each stage, we conducted pilot tests to revise the questions.

In Stage 1, the aim was to identify the initial design requirements for the digital storytelling application. This was achieved through a comprehensive methodology that included six online focus groups, one face-to-face focus group, and online one-on-one interviews. Participants involved 31 individuals with MCI, 13 care partners (with sessions lasting 90 minutes each), and 10 therapists (with sessions lasting 60 minutes each). The outcome of this stage was the establishment of crucial initial design requirements.

Moving on to Stage 2, the primary objective was to generate and visualize design requirements for the application. This stage encompassed eight workshops featuring brainstorming, card sorting, and quick prototyping activities. Four co-design sessions were held at Jingshilaonian from February to March 2023, with participants including 12 people with MCI and four care partners, each session lasting 90 minutes. The outcomes of Stage 2 were not only improved design requirements but also a designed prototype that visually represented the collaborative efforts. Based on these design requirements, the research team designed the mock-up of a digital storytelling application by employing the JS Design (https://gees.ai) to craft the interface. JS Design is a web-based interface design tool that has gained a significant following among professionals in user interface user experience design, graphic design, and web development.

Finally, independent usability testing was conducted in May 2023. In Stage 3, the aim was to evaluate the design requirements implemented in the digital storytelling application and identify the design recommendations. The methods employed to evaluate the prototype included a cognitive walkthrough^44^ facilitated by author DZ and interviews. Participants involved 12 people with MCI and five therapists, with each session lasting 45 minutes. The outcomes of this stage were the final design requirements, ensuring that the digital storytelling application met the intended goals through a rigorous testing and an evaluation process.

### Data collection

Throughout various phases of our research, qualitative data was collected systematically through interviews, sketches, and focus group discussions and documented meticulously via audio recordings. In the inaugural stage (Stage 1), the focus was to accrue comprehensive insights into the facilitator of social participation. The facilitator (first author) has extensive experience and training relevant to the study. They have conducted interviews, focus groups, and co-design workshops, all of which are essential methods for gathering qualitative data. Additionally, the researcher has a background in psychology and holds a master's degree in the field, which provides a strong foundation for understanding participants’ cognitive and behavioral aspects, especially in studies involving vulnerable populations like those with MCI. This combination of practical experience in qualitative research methods and a solid educational background in psychology equips the researcher with the necessary skills to design and execute the study effectively. The data collection took place in the meeting room of a partner organization, which was conveniently located close to the majority of participants. During the data collection process, a research assistant assisted by recording data, distributing, and collecting documents. And the facilitator and research assistant coded the transcript. In Stage 1, we conducted focus groups with people with MCI and their care partners, while therapists participated in one-on-one interviews. Specifically, there were a total of 10 one-on-one interviews conducted with therapists. Each focus group session with persons with MCI and care partners lasted approximately 90 minutes, providing ample time for in-depth discussion and feedback. Progressing to Stage 2, we conducted focus group discussions to discern and analyze the prioritized facets of social participation. Stage 3 encompassed dynamic participant involvement in brainstorming activities, wherein generated sketches and discussions ensued regarding potential features for a digital application. Finally, in Stage 3, participant feedback was methodically obtained through self-reporting during qualitative evaluation sessions and structured interviews.

### Data analysis

Reflexive thematic analysis was employed to distil and refine the design requirements identified in the three stages of the study. This qualitative analysis method allows for a systematic exploration of patterns, meanings, and insights embedded in the data collected.^45,46^ In Stage 1, adhering to COREQ guidelines,^47^ focus groups, interviews, and feedback from qualitative evaluations were recorded, transcribed, and reviewed for accuracy. The transcriptions were reviewed and evaluated by caregivers. Data saturation was achieved in our research when no new themes or insights emerged during the interviews and focus groups. This indicates that the data collected was sufficient to thoroughly explore the research questions, ensuring the reliability and completeness of the findings. The researchers cross-checked the transcripts to ensure a thorough understanding of the data. Chinese transcripts underwent care partner scrutiny and were translated into English with DeepL (https://www.deepl.com/translator). English transcripts underwent additional scrutiny by proficient English readers and translators, after which, the codes were reorganized into themes. NVivo was used during the analysis stage. Two transcripts, characterized by rich information content, were selected for detailed analysis, with two researchers proposing thematic codes. Subsequent collaborative discussions refined the final code headings and descriptions, employing labels such as “Stage 1-G2-P2” to differentiate between various groups and participants.

To identify the main themes within the storytelling application, our study utilized a rigorous process involving multiple iterative stages. The primary goal was to identify design requirements that accurately reflect the needs and preferences of people with MCI. Digital storytelling interventions utilize physical prompts to facilitate the recollection of past actions, events, and emotions. By integrating insights from a comprehensive literature review on digital storytelling interventions, we identified four key features necessary for effective digital storytelling applications: Identified Preferred Storytelling Themes, Story Material Generation, Memory Retrieval, and Story Sharing. Previous studies have demonstrated that these elements are crucial for enhancing the user experience and engagement in digital storytelling, particularly for individuals with cognitive impairments.^6^ Effective storytelling applications must support users in identifying and focusing on themes that resonate with their personal experiences and interests, helping to organize and structure their narratives and making the storytelling process more meaningful and engaging.^48^ Facilitating the generation of story materials is also essential for users with MCI, as the application should provide tools and prompts to help users collect and create relevant materials, such as photos, videos, and text, which are crucial for building their stories. Enhancing memory retrieval is another critical aspect, as tools that assist users in recalling and organizing their personal memories enable them to access and share their experiences more effectively, supporting cognitive engagement by helping users connect with their past. Lastly, the ability to share stories with others is vital for fostering social interaction and engagement, allowing users to connect with their social circles and enhancing their sense of community and belonging. Sharing stories can also provide opportunities for feedback and support, further enriching the storytelling experience. These themes were identified as foundational for developing a digital storytelling application that is both user-friendly and effective in promoting social participation and cognitive engagement among people with MCI, addressing their unique needs and preferences comprehensively.

Thematic analysis was applied to transcripts and findings from six online focus groups, a face-to-face focus group, and one-on-one interviews. Through an in-depth exploration of emerging themes, patterns, and nuances, initial design requirements were identified and organized into coherent themes reflecting participants’ perspectives. The identification of design requirements involved three iterative stages: (1) initial identification: in the first stage, we conducted an initial thematic analysis to identify potential themes based on preliminary data. This initial analysis provided a broad understanding of the participants’ needs and preferences. (2) Feedback and adjustment: in the second stage, these preliminary themes were presented to participants for feedback. This stage involved design workshops where reflexive thematic analysis was applied to the outcomes of brainstorming, card sorting, and quick prototyping activities. Participants’ insights were used to adjust and refine the themes, revealing thematic patterns related to improved design requirements and prototype conceptualization. (3) Final refinement: the third stage involved a final round of thematic analysis, incorporating all previous feedback and adjustments. Reflexive thematic analysis examined the results of qualitative prototype testing, including cognitive walkthroughs and interviews, synthesizing final design requirements aligned with participants’ needs. Throughout these stages, we used Braun and Clarke's guidelines for thematic analysis to maintain a systematic and rigorous approach. The iterative reflexive thematic analysis allowed continuous refinement and evolution of design requirements, fostering a dynamic and responsive approach to the iterative design process. Reflective insights gained contributed to a holistic understanding of evolving design requirements, ensuring a robust and user-centered digital storytelling application.

## Findings

### Design requirements for a storytelling application from Stage 1

#### Identified preferred storytelling themes

Connecting with like-minded old and new friends: Group-member cohesion linked strangers quickly. The therapist highlighted that every person with MCI has many attributes, such as hometown, job, and hobbies. These attributes serve as a unifying force within a group of individuals. Beyond the direct augmentation of the activity experience, engagement with peers afflicted by MCI facilitates reciprocal exchange. One participant explained a heightened sense of relaxation because they feel they are not alone.

Because he from this activity, including the family, will feel a breath of relief, or feel a little bit more relaxed, or he sees similar people, he is not so depressed. If he sees someone worse, he will be even less depressed, saying that there are people worse than my father and mother who have confidence. [Stage 1—Therapist 7]

#### Story material generation

Empowering team captains to drive activity enthusiasm and participation: Peer support may also enhance social participation. As a few participants (*n* = 2, 6.4%) and some therapists (*n *= 6, 60%) discussed in the social activity, a leader motivates those with MCI and brings cohesion to the team.

I think that the group inside, ah, there should be so many key figures to drive the group or can be said to be the leader of these people gathered together. Also, play a role like a leader. To enable him to better integrate. [Stage 1—Therapist 8]

Enhance retainment capabilities and compensate for weakened ones: Some of the therapists (*n* = 2, 20%) explained that many people with MCI have enough ability to attend social activities, albeit with some impaired abilities. One of the participants with MCI stated:

For example, I see a doctor with medication, see then also go, and now forget, now I cannot recall, but thinking it can still, he is unable to think slowly think up, in the development of it unfold it to see a problem, how do I see? How to evaluate? Why the future development towards my thinking, understanding or strong. [Stage 1-FG1-P3]

#### Memory retrieval

Creating a dynamic and interactive atmosphere: Some people with MCI (*n* = 4, 12.9%) reported that they are willing to accompany teenagers, children, or care partners, with passion. These people are energetic, creative, youthful, and friendly. Some therapists (*n *= 6, 60%) explained that connecting with their grandchildren may help some people with MCI to feel more youthful.

It can have some different groups that can care for them, and then this kind of group, this kind of group will have an impact with them, that is, it's lively and youthful, on the participation or interaction of volunteers. [Stage 1—Therapist 1]

Table 2 delineates the features and design requirements incorporated into the digital storytelling application to augment the user experience. Notably, it enables users to “connect with like-minded new and old friends” by leveraging identified preferred storytelling themes. In the realm of narrative material generation, the design strategically empowers team captains to drive enthusiasm and bolster participation, concurrently enhancing and compensating for any diminished faculties. The memory retrieval aspect is distinctly characterized by its emphasis on creating a “dynamic and interactive atmosphere” to foster engagement and connection.

**Table 2.** Initial storytelling application design requirements from Stage 1.

Features	Design requirements
Identified preferred storytelling themes	Connect with like-minded new and old friends
Narrative material generation	Empower team captains to drive enthusiasm and participation in activities
	Amplify retained capabilities, compensate for weakened ones
Memory retrieval	Creating a dynamic and interactive atmosphere

### Design requirements for storytelling application from Stage 2

#### Identified preferred storytelling themes

Inspiring narratives, selecting themes that evoke positive emotions: Themes serve as the inception of storytelling, offering individuals the opportunity to explore and choose themes that bear personal significance, thereby nurturing a personalized and captivating storytelling journey. One therapist suggested that:

From an older adults perspective, they prefer reminiscing about their real stories. Having a variety of themes to choose from not only acts as a starting point but also enhances the overall activity. Moreover, these story themes should be easily accessible right on the homepage, making it intuitive for them to pick and engage with. [Stage 2—Therapist 5]

#### Story material generation

Unlocking memories, a step-by-step journey through key questions: Participants with MCI have expressed that when initially confronted with a story theme, they frequently encounter uncertainty about how to begin. They proposed the inclusion of guiding questions to aid in prompting their thoughts and recollections.

You know, sometimes when we get a story theme, it can be a bit overwhelming, not knowing where to start. So, I hope to make things a bit easier for us. Like providing us with some questions to help kickstart our thoughts and memories. It's like giving us a friendly guide, making the whole creative process more relaxed and enjoyable. We hope these questions can be a good starting point for our storytelling journey, adding more depth and meaning to each theme. [Stage 2—G3-P2]

Pre-prepared hints, the key to effortless story sharing: In elevating the storytelling experience, we have proactively created keyword prompts and cheat sheets, providing a safety net for potential challenges. This foresight allows for seamless narrative guidance during story sharing, ensuring more coherent and vivid storylines. Such support facilitates vibrant narrative creation, allowing individuals to effectively capture the essence of their memories and experiences:

To make our storytelling smoother, we could get these cheat sheets ready with keywords, just in case things get a bit tricky. So, when we're sharing our stories, we can easily steer the tale, making sure it flows nicely and paints a vivid picture. [Stage 2—G1-P2]

#### Memory retrieval

Exemplary progress, continuous motivation and community connection: A majority of participants (*n* = 9, 75%) expressed admiration for older peers who exhibit confidence in participating in diverse activities. Interacting with self-assured older people offers a new perspective on aging and life experiences, inspiring them to confront challenges, surmount obstacles, and adopt a positive mindset. Observing the progress and achievements of others becomes a source of inspiration and encouragement for people with MCI, fostering a sense of community and shared accomplishment.

They provide me with strength and inspiration, showcasing passion and a positive approach to caring for others. This makes me believe that I can still lead a fulfilling and meaningful life, even in difficult circumstances. Seeing the progress of others also adds to this sense of community and motivates me to keep moving forward. [Stage 2-G3-P3]

Hints to provide confidence in memory retrieval: The presentation feature facilitates memory retrieval by offering guidance for people with MCI. This empowers users to navigate through their memories with confidence, transforming the storytelling process into a journey of self-reflection and recollection.

The cheat sheet will be my memory aid, offering tips tailored for me. I'll confidently navigate through my own memories, transforming the storytelling process into an anticipated journey of self-reflection and recollection. [Stage 2—G4-P1]

#### Story sharing

Beyond the story, sharing with a broader audience: Digital storytelling promotes meaningful social interaction and connection by affording users the capability to share their stories with wider audiences. One therapist stated:

Sharing with more friends promotes engagement not only within the application but also in social activities outside the home, fostering a sense of community and participation. [Stage 2—Therapist 1]

According to the information in Table 3, users can engage in uplifting narratives by choosing themes designed to evoke positive emotions. Story material generation unfolds as a guided journey, unlocking memories through a sequential exploration of key questions. The memory retrieval feature is characterized by the provision of hints aimed at instilling confident recollection and is complemented by pre-prepared cheat sheets, for effortless story sharing. The application goes beyond individual stories by encouraging users to share their stories with a wider audience. The digital storytelling application features a unique “Personal Memory Vault” that helps users tap into a wellspring of stories during the narrative material generation process. The application's design places particular emphasis on memory retrieval, through the concept of “Exemplary Progress” which is intended to foster a sense of community and connection throughout the storytelling journey.

**Table 3.** Storytelling application design requirements from Stage 2.

Features	Design requirements
Identified preferred storytelling themes	Inspiring narratives: choose from story themes listed on home page that evoke positive emotions
Story material generation	Unlocking memories: a step-by-step journey through key questions
	Prepared hints: the key to effortless story sharing
Memory retrieval	Exemplary progress: continuous motivation and community connection
	Hints in hand, confidence in memory retrieval
Story sharing	Beyond the tale: share it with more

### Qualitative testing results from Stage 3

**Figure 2.**
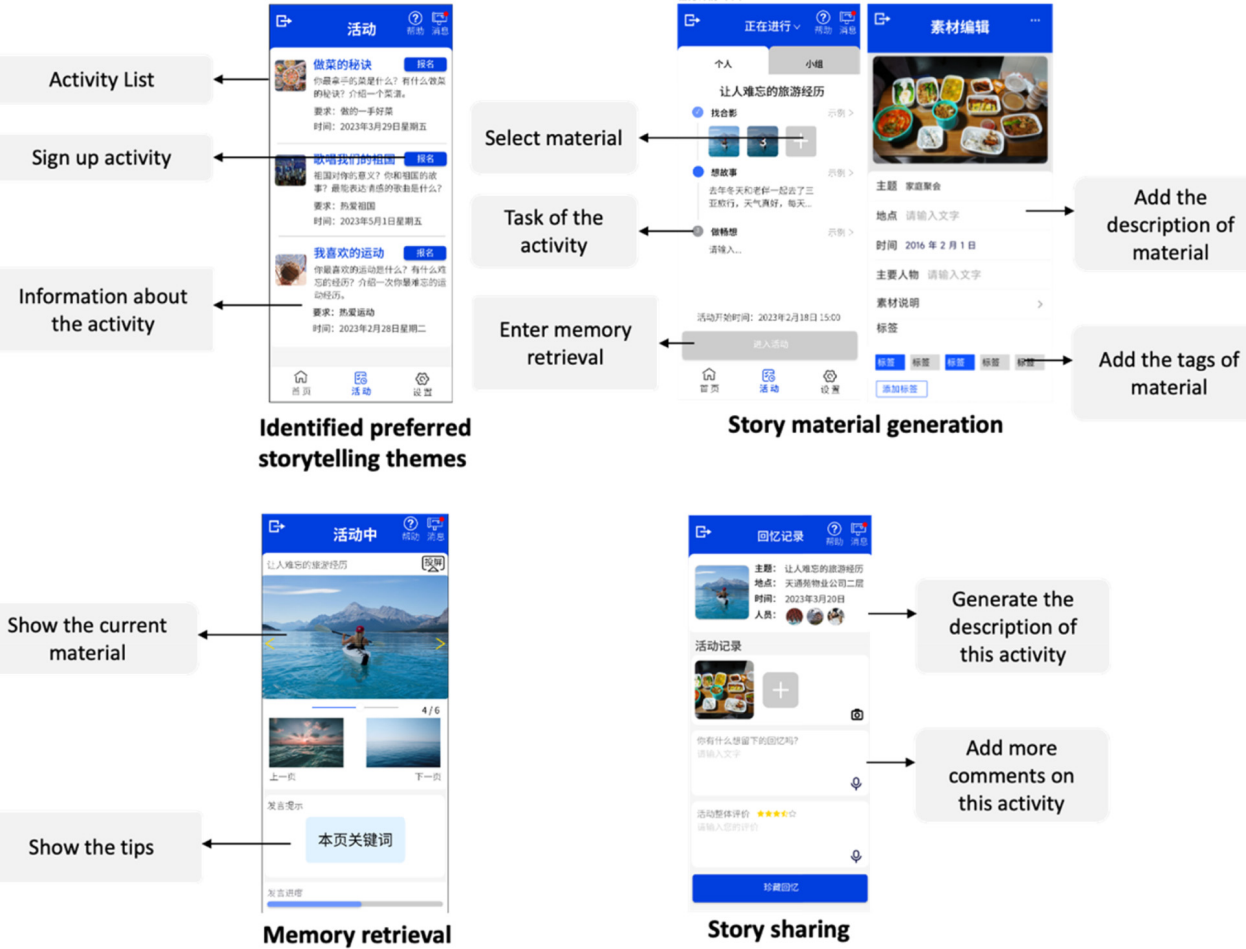
Key components of the prototype.

In developing our digital storytelling application, we have meticulously crafted an interactive prototype, tailored to meet the specific design requirements identified in our research. Utilizing JS Design, our team has created engaging and user-friendly mock-ups that not only embody the core functionalities but also provide an immersive interactive experience. This prototype encapsulates key elements such as identified preferred storytelling themes, tools for story material generation, features aiding in memory retrieval, and seamless story sharing capabilities (see Figure 2). Each aspect of the prototype has been thoughtfully designed to enhance the storytelling experience, ensuring it is not only intuitive but also deeply resonant with the needs of people with MCI. Users sign up by selecting their preferred story themes, preparing corresponding images and video materials as prompted, and choosing relevant tags and content for these materials. They can also arrange the order of the different materials. Once the activity starts, they engage in story recollection. Users can see the corresponding prompts they have written, ultimately generating a record of the activity.

The participants’ subjective experiences highlighted several positive impacts of the activity. They reported making new friends, which expanded their social circles. Many found joy in reminiscing about past enjoyable moments and felt a renewed sense of pleasure. The activity also provided them with opportunities to speak out, boosting their confidence and communication skills. Additionally, participants experienced significant improvements in their memory, which helped them recall daily details more effectively.

For example, one participant shared, “We chatted away, made new friends. Without this activity, I wouldn't have met everyone, you know” [P1]. Another participant expressed the joy of reminiscing, saying, “We were reminiscing about fun things from the past, which I'd forgotten, but remembering brought back all those lovely moments [P9]. The activity also created opportunities for speaking, as noted by a participant, “We never had the chance to speak out before, never said a word in such gatherings, now I'm brave enough to” [P3]. Another participant found joy in remembering past times, “In remembering, I've found the joy of past times, especially chatting away with all of you” [P6]. Furthermore, improvements in memory were highlighted, with one participant stating, “My memory's better now; used to forget the next line after just saying one, or what I was after in the kitchen” [P4].

Table 4 below provides a concise summary of the feedback received from participants during the Stage 3 interviews of the digital storytelling application study. The table is organized into four key areas based on the interview questions: features participants liked, and disliked, difficulties encountered, and suggestions for improvement. Each section captures specific participant feedback, reflecting their experiences and preferences, and offering actionable insights for refining the application design. For instance, participants expressed a preference for the story material generation feature, particularly appreciating the ability to tag and organize personal materials. However, they disliked the internet search functionality and faced challenges with the initial sorting mechanism due to unclear instructions. Participants suggested prioritizing recent activities in the registration process and requested more intuitive sorting methods and clearer guidance. These insights will guide targeted refinements to the application to better meet user needs and enhance overall usability.

**Table 4.** Participant feedback summary from Stage 3 interviews.

Question of Study 3	Participant feedback
Features liked	- Story material generation: Appreciated for ease of use, especially with tagging and organizing materials. - Face-to-face interaction: Liked the natural flow of conversations without the “raise hand” button.
Features disliked	- Internet search functionality: Disliked the requirement to use the internet for material uploads; preferred direct use of personal materials. - Sorting materials: Found the initial sorting mechanism challenging due to unclear instructions.
Difficulties encountered	- Sorting and organization: Struggled with the initial sorting process and found it not intuitive without clear guidance. - Editing permissions: Experienced confusion with editorial permissions during activity registration and material generation.
Suggestions for improvement	- Story material generation: Suggested adding more intuitive sorting methods like “drag-and-click” and making instructions more visible. - Activity registration: Recommended prioritizing recent activities and registration details for easier access. - Material sorting: Requested clearer instructions and support for multiple sorting methods. - Upload process: Suggested removing the internet search function to streamline uploading personal materials.

Table 5 outlines key features, their corresponding design requirements, and the associated cognitive walkthrough tasks for the digital storytelling application. Identified preferred storytelling themes, such as inspiring narratives, are linked to specific actions in the activity interface, emphasizing the importance of positive emotions. The story material generation involves tasks like uploading pictures and organizing materials, ensuring a step-by-step journey through key questions. Memory retrieval is facilitated by features like entering speech mode and finding screen projection buttons, promoting continuous motivation and community connection. Story sharing is enhanced with options like inserting a group photo after the event, extending the impact beyond the tale.

**Table 5.** Cognitive walkthrough tasks and design recommendations.

Features	Cognitive walkthrough task	Design recommendations
Identified preferred storytelling themes	Enter the activity interface Switch the view of the activities	None
	Browse registration activities Sign up for activities and add groups	-Display activity registration initially, followed by recent activities
Story material generation	Upload pictures New materials Enter my materials Change the sequence of materials View task progress	-Allow easy switching and searching for images -Delete the option of “Upload material through Internet” -Provide operating instructions and make the font color of the instructions more eye-catching -Provide support for two sorting methods: click and drag
	Add material description Add material tags	-Integrate the image library and add tags
Memory retrieval	Entering speech mode Find the screen projection button	-In face-to-face discussions, there is no need to set up a “raise hand” button for inquiries
	Find speech prompts	None
Story sharing	Insert a group photo after the event	None

The cognitive walkthrough tasks outlined in the table serve as a comprehensive guide to navigating and interacting with the digital storytelling application, ensuring a user-friendly and intuitive experience. For identified preferred storytelling themes, users are prompted to enter the activity interface and switch the view of activities, empowering team captains to drive enthusiasm and participation by browsing and signing up for activities. In the story material generation phase, users are guided through tasks such as uploading pictures, adding new materials, entering personal materials, changing the sequence of materials, and viewing task progress. Prepared hints for effortless story sharing involving adding material descriptions and tags, enhancing the overall storytelling process. Memory retrieval tasks include entering speech mode and finding the screen projection button for exemplary progress, providing users with hints and boosting confidence in memory retrieval. Finally, for story sharing, users are encouraged to go beyond the tale by inserting a group photo after the event, fostering a sense of community and extending the impact of shared experiences. The cognitive walkthrough tasks collectively contribute to a holistic and user-centered design approach for the digital storytelling application.

#### Identifying preferred storytelling themes

Establishing an activity registration setup has elicited specific user concerns, as participants have expressed their need for enhanced clarity and accessibility throughout the registration process. The imperative to streamline activity registration steps and optimize the presentation of pertinent details has been highlighted by users, aiming to facilitate a seamless, efficient user experience. A participating therapist stated:

When I'm signing up for something, I just want to see the details first, not scroll through a bunch of old activities. [Stage 3—P5]

Users encountered issues with the activity registration sequence and comprehension of editorial permissions. To ameliorate these features, we prioritized displaying the activity registration first, and prominently showing recent activities is recommended.

#### Narrative material generation

Additionally, the sourcing of materials online has raised questions and preferences among users.

- Adding tags to each piece of material: Participants have expressed difficulties in identifying and locating previously uploaded materials and viewing associated images, which highlights the need for enhanced organization and a user-friendly retrieval mechanism in the application's gallery:Tag my photos and let me switch and search easily. [Stage 3—P6]

Can we organize photos, like by time, location, and people? [Stage 3—P8]

**Figure 3.**
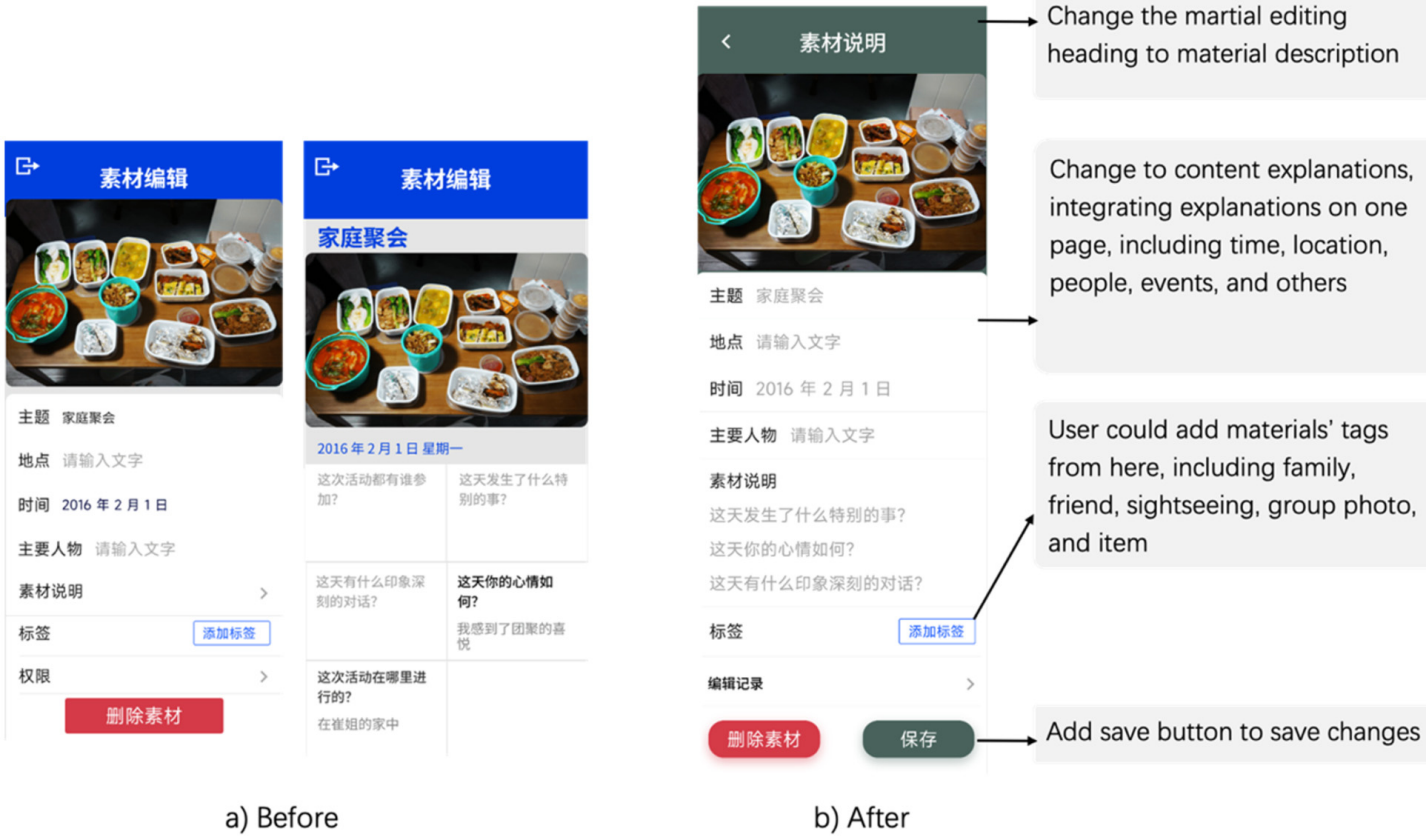
(a) Inability to understand editing functions and permissions and (b) re-designed interface.

As Figure 3 shows, the heading “Martial Editing” has undergone modification to “Material Description” for enhanced clarity and comprehension. As part of this development, all material descriptions have been integrated conveniently into a single page, encompassing important details such as time, location, people, and events. Such a consolidation ensures easier access and offers a comprehensive overview of the content. Users can now add tags, including family, friends, sightseeing, group photos, and items. Introducing a new “Save” button enhances the user experience, allowing changes to be preserved during the content uploads. These improvements aim to enhance user satisfaction and optimize functionality for a more effective storytelling experience.

Easy to arrange materials, with instructions: The lack of an effective method of material arrangement has emerged as a usability concern, prompting users to request clearer instructions and intuitive sorting mechanisms, including: ‘drag-and-click’, for an enhanced user experience:Sorting was a bit tricky. Put some big instructions and let me sort by dragging or clicking. Easy peasy. [Stage 3—P8]Let me sort with drag and click; keep it easy. [Stage 3—P11]

**Figure 4.**
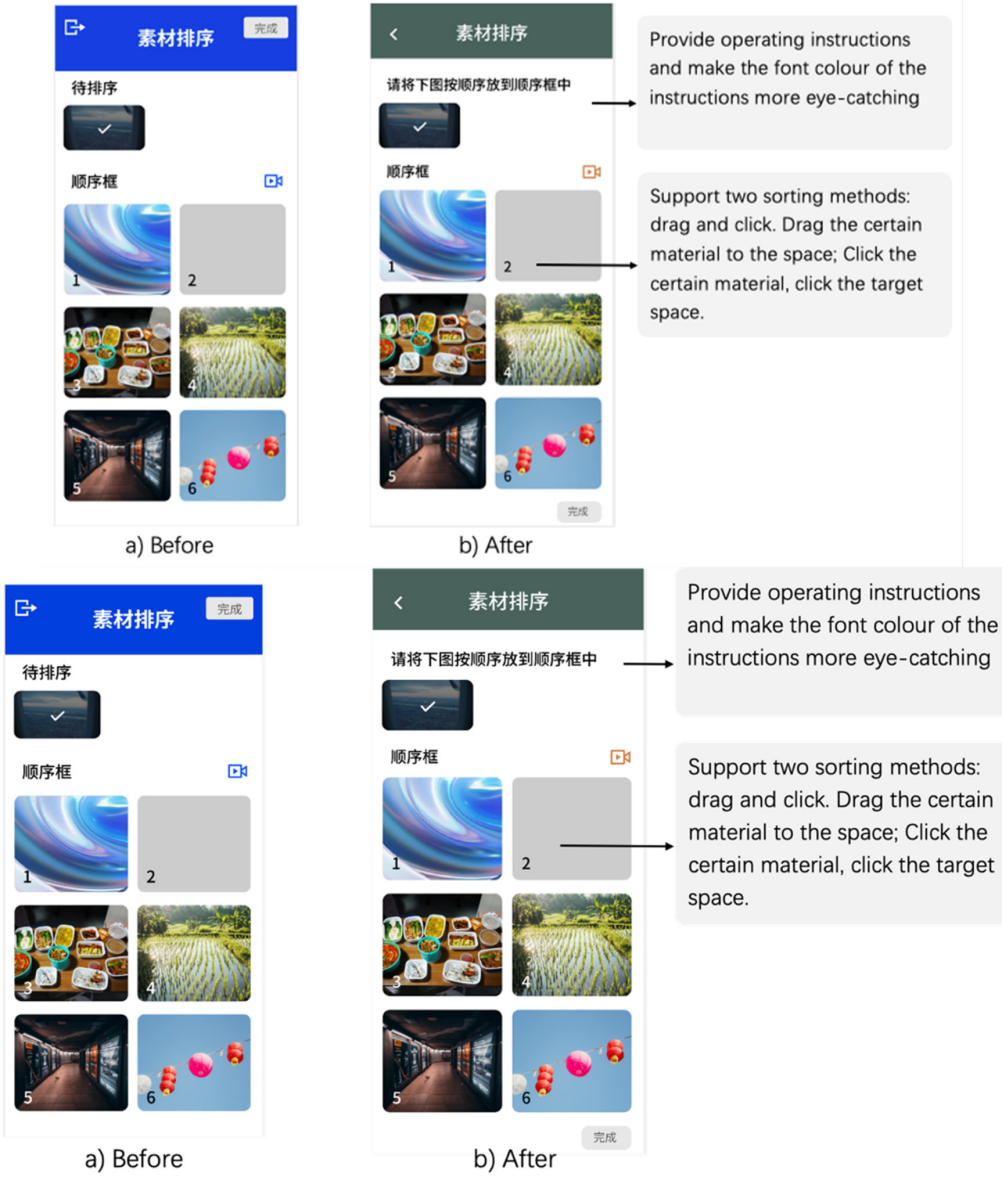
(a) Inability to sort content, (b) redesigned interface.

Figure 4 illustrates the users’ desire to modify the sequence of selected materials in a presentation. Explicit and succinct operating instructions have been incorporated, ensuring effective navigation of the application. Furthermore, two sorting methods have been introduced: drag-and-click. Users can either drag specific content to the desired location or click on the material and then click on the target space for placement. These improvements are directed towards enhancing software intuitiveness and accessibility, fostering a seamless and enjoyable storytelling experience for users.

**Figure 5.**
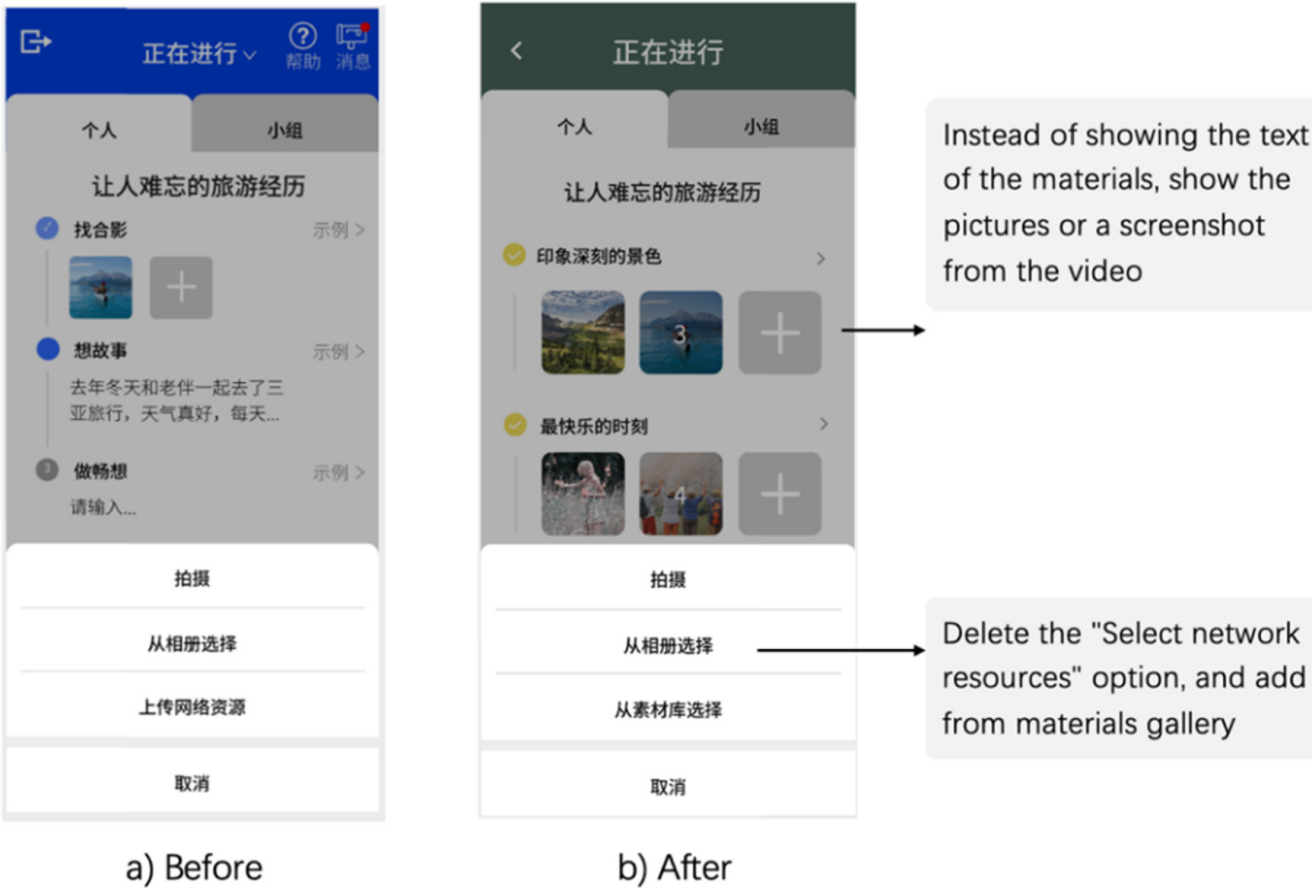
(a) No internet search function requirement, (b) redesigned interface.

Users have articulated preferences to streamline the upload process by eliminating requirements to source materials online. This manifests an inclination for a simplified and direct approach to uploading content within the digital storytelling application (sees Figure 5):

Internet? Nah, just let me upload stuff without that. Get rid of it. [Stage 3—P6]

Figure 5 highlights a departure from text-based content. To streamline the process, the “Select network resources” option was replaced with a direct selection option from the gallery. This change ensures a smoother and more efficient content selection process, facilitating users in accessing and utilizing their chosen visuals for storytelling. The objective of the updates is to enhance the application's visual stimulation and user-friendliness.

#### Memory retrieval

Maintaining face-to-face interactions: Users have emphasized the perceived redundancy of a ‘raise hand’ button during face-to-face discussions, expressing a preference for more natural and spontaneous interactions that eschew the need for formalized cues. This insight accentuates the significance of aligning application features with real-world social dynamics:

That ‘Share the screen’ thing is tiny. Make it big! And seriously, seriously, there is no need for a ‘raise hand’ when we're talking face-to-face. [Stage 3—P5]

Users have observed that face-to-face discussions, especially in offline meetings, obviate the need for a dedicated button to ask questions. Disabling the “raise hand” function for in-person meetings streamlines the interaction process.

Table 6 encapsulates valuable insights derived from qualitative user testing results wherein participants interacted with the application. Each row represents a specific feature: user-identified design requirements, verbatim quotes articulating concerns and suggestions, and the participant's identification number. This user-driven feedback forms an essential foundation for refining and enhancing the user interface and functionality of digital storytelling applications, thereby ensuring optimal usability and effectiveness.

**Table 6.** Final storytelling application design requirements and recommendations from Stage 3.

Features	Design requirements
Identified preferred storytelling themes	Prioritize the display of recent storytelling activities at the forefront
Story material generation	Add tags to each material
	Easy to sort story materials with instruction
	Use your own materials
Memory retrieval	Maintain a sense of face-to-face interaction
Story sharing	[No new design requirements to be received]

## Discussion

**Figure 6.**
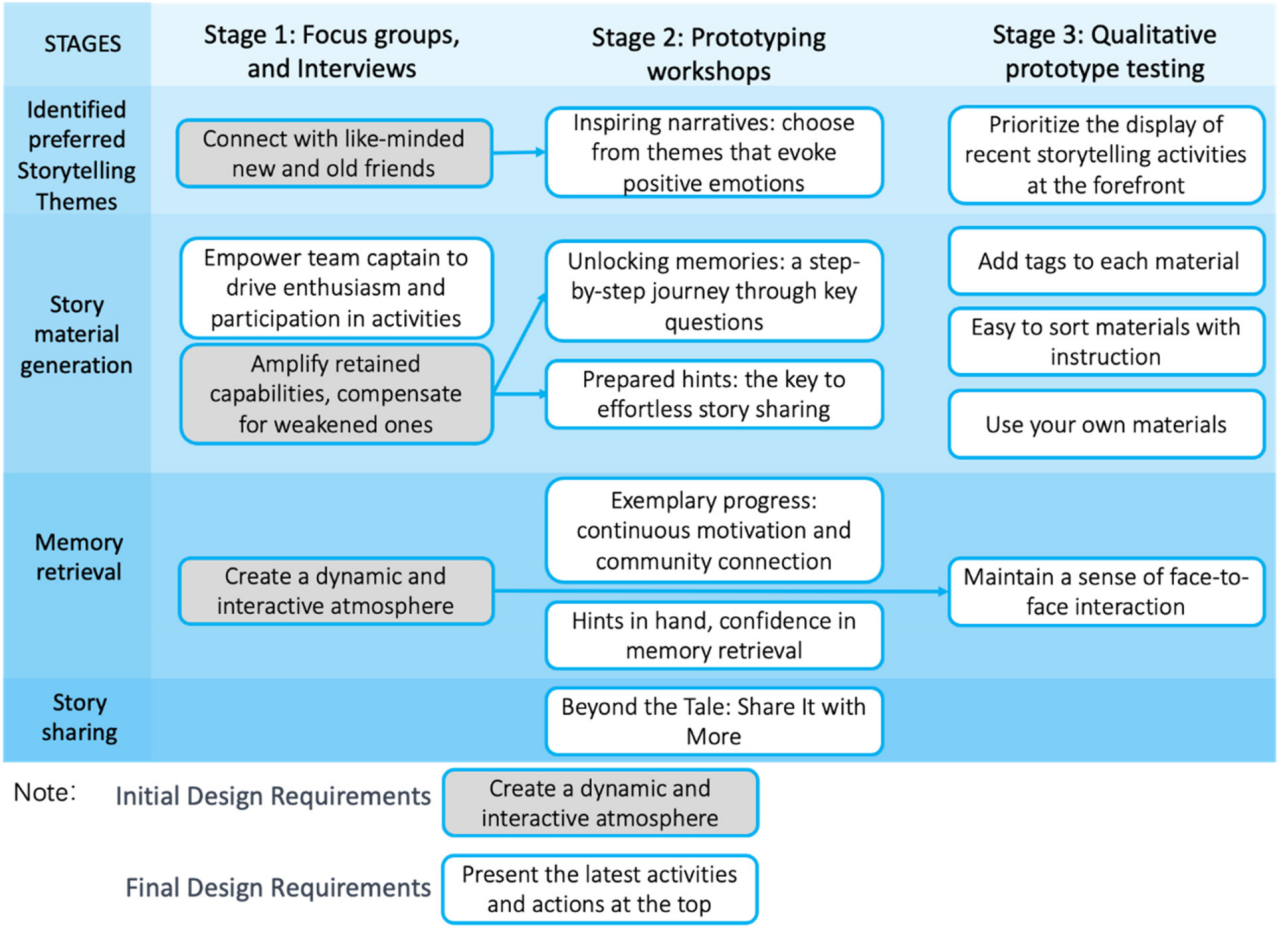
Twelve design requirements from all three stages (three design requirements were revised).

This study aims to delineate the design requirements for a digital storytelling application dedicated to supporting people with MCI in capturing recent, positive memories, specifically fostering social connections and camaraderie. Employing a three-stage research methodology, we gathered and synthesized design requirements for key features (see Figure 6), identified storytelling themes, generated narrative materials, facilitated memory retrieval, and enabled seamless dissemination. Each stage provided insights into the needs of our target users. Initial design requirements were broad and conceptual, establishing a foundational framework for later refinements. For instance, the desire for a dynamic and interactive atmosphere emerged in the first stage. The preliminary design concept transitioned into more concrete directives as the research progressed. Notably, the third stage underscored the imperative to “maintain face-to-face interaction,” substantiating earlier conceptual notions. Evolution from abstract ideals to tangible specifics refined our understanding and propelled the design process, ensuring alignment with user needs. The dynamic interplay between initial and refined design requirements mirrors our iterative approach, wherein user feedback informs our pursuit for an optimal solution.

Across the three-stage research process—comprising focus groups and interviews (Stage 1), prototyping workshops (Stage 2), and qualitative prototype testing (Stage 3)—key design requirements for various functionalities emerged. Regarding preferred storytelling themes, the emphasis was on narratives that inspire positive emotions. In narrative material generation, empowering team captains instilled enthusiasm, unlocked memories through targeted questions, and facilitated seamless story sharing via prepared hints. Memory retrieval underscored sustained motivation and fostered community connection to bolster confidence in the process. Story sharing subsequently sought to transcend mere narration by enabling broader dissemination. Throughout these stages, considerations such as prominently featuring the latest activities, incorporating tags, and preserving a sense of face-to-face interaction were integrated.

### Refined design requirements from all three stages

Our study aimed to design a digital storytelling application that facilitates group-based storytelling. Participants were encouraged to prepare their individual stories in advance, which were then shared and discussed in a group setting. There was consistency between the data presented and the findings. The study effectively used the data collected from interviews and focus groups to support its conclusions. The themes and insights derived from the data were clearly linked to the findings, demonstrating a logical progression from evidence to interpretation. This alignment ensures that the conclusions drawn are both reliable and well-supported by the data. This approach ensures that while the initial preparation is done independently, the primary focus remains on group-based interaction, enhancing social participation and cognitive engagement among people with MCI.

#### Story material generation

The digital storytelling application facilitates the storytelling process for people with MCI who share common interests in narrative themes. Diverging from group storytelling formats like the WeChat-based life review program^22^ that operates with predetermined materials and allows group story creation,^7^ this application offers preset content prompts to encourage independent story sharing by people with MCI. In the context of material generation, it promotes a self-directed collection of relevant materials in daily life, expanding opportunities for reminiscence and concurrently evoking positive emotions during the process. This approach contrasts with prevalent research practices employing pre-prepared materials,^21^ often involving care partners or volunteers in multimedia content editing on behalf of older adults.^49^ In this context, people with MCI assume the role of directors, seeking assistance in story development, which aligns with the consideration of reduced digital literacy levels. However, these risks diminish the sense of achievement post-reminiscence and necessitate additional human resources, such as tech-savvy care partners or volunteers,^10^ potentially limiting participation due to inadequate support. Few interventions advocate for independent editing by older adults post-training unless featuring fewer customization features, such as Moon & Park's digital memory retrieval application,^12^ which incorporates pre-set images and allows people with MCI to upload photos from their digital galleries.

Beyond material collection, the sequencing of materials significantly influences the subsequent memory retrieval activity. Consequently, the digital storytelling application supports people with MCI in arranging the display sequences of materials, akin to the OurStory application.^13^ Recognizing that memory retrieval activity may necessitate heightened stimuli; deliberate sequencing aims to optimize positive memory activation, deviating from some storytelling applications that present materials randomly.^12^ Therefore, the focus of the digital storytelling application lies in presenting a multimedia content in a manner that maximizes the activation of positive memories, thereby facilitating recollection.

#### Memory retrieval

People with MCI have the ability to selectively employ memory cues during storytelling, as opposed to those diagnosed with dementia, who may require life-logging technologies to capture and extract memory cues automatically.^26^ The shared memories act as conversational prompts, fostering meaningful connections among peers. Corresponding with the TIM board game, this research posited that social connections may be enhanced by narratives and reflections on relational citizenship.^50^ The key to a successful storytelling program is to evoke positive emotions during the shared experience and encourage participants to discuss and reflect on their experiences. Research shows that while generic topics may elicit similar memories and reactions among different people, personal subjects act as a memory test for names and details of family events, which can boost storytelling and social memory.^51^ If the story is complete, older adults may verbalize and explore it in more depth.^52^ These adults share their stories based on an edited video and script. Randomly showing visual materials appears to be the most popular method due to its efficiency.^12,53^ Some applications reveal a sequence of individual preferences,^54,55^ and in one instance, an application displays photographic materials on a timeline.^10^ Visual cues, such as images, video clips, and text, are the most popular media formats designed to stimulate verbal memory retrieval.^11^ Music also plays an important role during the reminiscing stage^54^ as it can evoke and trigger related memories. Multiple technology platforms can engage older adults simultaneously or consecutively, such as television displays showing video content, radios playing music, telephones recounting poems or songs, and treasure boxes presenting selected objects to aid remembrance and promote tactile stimulation.^56^ Retriggering processes could instigate further memory retrieval, as in group-based reminiscence therapies, where comments from others formed new talking points.^21^

In facilitating the recollection and sharing of recent positive memories, the digital storytelling application serves to elevate the social confidence of people with MCI within group settings. Participating in memory groups has been demonstrated to correlate with an increased utilization of compensatory strategies and heightened memory-related self-confidence.^57^ The ability to recount recent experiences and actively engage in discussions instils a sense of self-assurance, empowering individuals to participate actively and contribute meaningfully to social gatherings and activities. Moreover, participants expressed satisfaction with the duration of individual presentation time and self-expression during the group discussions, underscoring the intrinsic value of listening to stories shared by others. The act of hearing others’ accounts not only facilitates self-reflection but also inspires a diverse pool of revisited social activities.

#### Story sharing with others

Sharing recent memories through a storytelling application has been identified as a stimulus for dialogue, fostering active engagement in social interactions for people with MCI. Older adults may participate in reminiscing sessions or record memories for dissemination among family, friends and broader networks. While some interventions lack a clear structure or script during the memory retrieval process,^17^ others systematically record and share stories through the application^49^ or website.^21^ Notably, the choice of a sharing platform holds implications for privacy and safeguarding concerns, prompting the selection of WeChat as the primary platform, given its prevalence among friends and family members.

Moreover, participants expressed a lack of concern regarding the duration of individual sharing, as the activity holds intrinsic value in listening to others’ stories. The act of hearing narratives shared by peers allows individuals to reflect on their own lives and derive inspiration from a diverse array of social activities. Consequently, older adults may share their stories during reminiscing sessions or record reminiscences for distribution among family, friends, and extended networks. While some applications facilitate casual reminiscing without a defined structure or script,^17^ others aim to record and share structured narratives through the application,^49^ or website.^21^ Although sharing on a website extends the reach to a broader audience beyond immediate family and friends, this approach introduces privacy and safeguarding concerns.

Our study addresses a critical gap in the existing technology for people with MCI and dementia by developing a digital storytelling application designed for independent use. This innovation is significant because it reduces the reliance on care partners or tech-savvy helpers, which can often be a major barrier to technology adoption and daily use for people with MCI. By empowering people with MCI to use the tool independently, our application enhances their ability to engage in meaningful storytelling activities, thereby promoting cognitive engagement and social interaction.

#### Theoretical framework comparison

The design requirements and features of the digital storytelling application align with the principles of the International Classification of Functioning, Disability and Health (ICF) and Social Cognitive Theory (SCT). These frameworks emphasize the importance of cognitive, physical, and social factors in enhancing participation and functioning for people with MCI. In the ICF framework, activities and participation are supported by features such as Identified Preferred Storytelling Themes and Story Material Generation.

Inspiring narratives and prioritizing recent storytelling activities help users engage in meaningful activities, maintaining cognitive functions and social interactions, which are essential elements of participation as outlined by the ICF. Empowering users to generate their own story materials and providing tools to unlock memories facilitate active participation in cognitive tasks. Features like step-by-step memory unlocking and prepared hints ensure users can engage in these activities independently, promoting self-efficacy and reducing reliance on external help. Environmental factors in the ICF framework are addressed through the Story Sharing feature. Facilitating easy sharing of stories with others, both within the application and through broader platforms, enhances the social environment for users. Maintaining a sense of face-to-face interaction and using familiar platforms like WeChat supports users’ social connections and community involvement. For instance, while the WeChat-based life review program^22^ focuses heavily on social interaction, it lacks the emphasis on independent content creation seen in our application. Assistive technologies, such as adding tags to materials, easy sorting instructions, and the use of personal materials, simplify the storytelling process, aligning with the ICF's emphasis on the role of environmental factors in facilitating participation. Similarly, existing frameworks such as the TIM board game^51^ facilitate social connections through structured activities but do not address the technological barriers faced by people with MCI, highlighting the need for solutions that integrate assistive technologies to support cognitive and social engagement. Personal factors in the ICF framework are supported by features that empower and motivate users. Empowering a team captain to drive enthusiasm and continuous motivation through exemplary progress fosters a supportive social environment, enhancing self-efficacy by providing social support and recognition of achievements. Cognitive and emotional support features, like hints in hand for memory retrieval and prepared hints for story sharing, reduce cognitive load and boost users’ confidence in their abilities. These elements support emotional well-being and cognitive functioning, which are critical personal factors in the ICF model.

In the SCT framework, self-efficacy is enhanced by allowing users to choose from themes that evoke positive emotions, reinforcing their confidence in their storytelling abilities.^31^ Story Material Generation features, providing step-by-step guidance and prepared hints, support users in generating and organizing their materials independently, increasing their confidence in their ability to complete storytelling tasks successfully. Social support and outcome expectations are addressed through the empowerment of a team captain, fostering a sense of belonging and collective efficacy, and encouraging users to share their stories within a supportive community. This aligns with SCT's emphasis on the role of social support in achieving desired outcomes. Goal setting and behavioral change are supported by features like prioritizing recent storytelling activities, helping users set achievable goals and track their progress, and facilitating memory retrieval and organization through tools that structure their storytelling activities effectively.

By merging the principles from the ICF model and SCT frameworks, our design requirements emphasize not only the importance of social and cognitive engagement but also the need for technology to be accessible and empowering for users with MCI. By aligning these design requirements with the ICF and SCT frameworks, the digital storytelling application supports the holistic development of cognitive, physical, and social skills. This approach enhances self-efficacy, facilitates meaningful participation, and fosters a supportive social environment for people with MCI.

### Limitations

A notable limitation of our research lies in the exclusive design of the application for the Android platform, motivated by the predominant use of Android phones among a majority of our participants with MCI. Consequently, the findings may not fully represent the experiences of individuals using alternative devices (e.g., iPhones). To mitigate this limitation, subsequent iterations of the study will encompass the design and testing of the application on alternative platforms, thereby ensuring a more comprehensive understanding of user experiences across different devices. Another limitation pertains to the focal point of participants with MCI residing in the community during the co-design and qualitative evaluation phases. This emphasis on community-dwelling individuals stems from the acknowledgment of their intricate needs in terms of social participation support. In the *Limitations* section of this study, it is important to acknowledge that while the MoCA score range of 18–25 was used to define the diagnostic interval for MCI, the potential impact of participants’ educational levels on these scores was not fully accounted for. The MoCA protocol suggests adding one point to the total score for individuals with 12 years of education or fewer, to mitigate the risk of underestimating cognitive abilities in those with lower educational attainment. However, this adjustment was not consistently applied in our study, which may have led to misclassification of cognitive impairment severity, particularly among participants with varying educational backgrounds. This oversight could introduce bias in the diagnostic process. Future research should aim to incorporate educational adjustments in the analysis and interpretation of MoCA scores to ensure a more accurate and equitable assessment of cognitive impairment. Despite recognizing the potential benefits of storytelling activities for people with MCI in care centers, the study did not extensively explore the application's efficacy in such settings. Future research should investigate the feasibility and effectiveness of embedding the digital storytelling application in care center environments, thereby fostering a more thorough understanding of its applicability across diverse settings.

## Conclusions

In conclusion, this paper comprehensively outlines the requisite design parameters governing the entirety of the operational trajectory of a digital storytelling application. The primary focus pertains to addressing and alleviating cognitive challenges encountered by people with MCI. The identified design imperatives not only facilitate the creation of a comprehensive and engaging narrative but also underscore the significance of individual preparedness and concurrent communal dissemination. By emphasizing these aspects, our research makes a substantive contribution to the conceptualization and implementation of a user-centric solution tailored to enhance the storytelling experience for people with MCI. The resulting application shows promise as an invaluable tool for memory support and social aid; however, further research, including trials or qualitative studies, will be necessary to determine its effectiveness in these areas. Through an iterative investigative process, we identified preferred storytelling themes, characterized by an emphasis on positive emotional content, devised strategies to empower team captains in the generation of narrative material, and concentrated on optimizing memory retrieval and seamless story sharing. The development, progressing from abstract conceptualizations to tangible specifics, manifests our commitment to the refinement and customization of our methodology guided by user feedback and evolving insights. The emphasis on perpetual motivation, community integration, and a design philosophy centered around user preferences positions our solution strategically to meet the diverse needs and preferences of our target demographic. By incorporating considerations such as effective presentation of activities, facile categorization through tags, and the preservation of face-to-face interaction, our objective is to create a user-friendly, immersive platform that transcends mere functionality to enhance the overall user experience.

## Supplemental Material

sj-docx-1-dhj-10.1177_20552076241282237 - Supplemental material for Design requirements for a digital storytelling application for people with mild cognitive impairment (MCI) Supplemental material, sj-docx-1-dhj-10.1177_20552076241282237 for Design requirements for a digital storytelling application for people with mild cognitive impairment (MCI) by Di Zhu, Abdullah Al Mahmud and Wei Liu in DIGITAL HEALTH

sj-pdf-2-dhj-10.1177_20552076241282237 - Supplemental material for Design requirements for a digital storytelling application for people with mild cognitive impairment (MCI) Supplemental material, sj-pdf-2-dhj-10.1177_20552076241282237 for Design requirements for a digital storytelling application for people with mild cognitive impairment (MCI) by Di Zhu, Abdullah Al Mahmud and Wei Liu in DIGITAL HEALTH
