# Supplementary material for: Design requirements for a digital storytelling application for people with mild cognitive impairment (MCI)
Source: Digit Health. 2024 Sep 19;10:20552076241282237. doi: 10.1177/20552076241282237 (PMC11418240; doi:10.1177/20552076241282237)
Supplement: sj-docx-1-dhj-10.1177_20552076241282237 - Supplemental material for Design requirements for a digital storytelling application for people with mild cognitive impairment (MCI) [file sj-docx-1-dhj-10.1177_20552076241282237.docx]

**Stage 1 Focus Group**

- How long have you been diagnosed with MCI?
- Could you please tell me about your typical day? What do you usually do? (The daily routine exercises)
- What kind of things do you tend to do around your home (e.g., go to the food market, pick up grandchildren, clean, cook, walk, square dance, manage finances)?
- Could you please tell me about your social connection circle?
- How do you usually connect with your family and friends?
- What kind of technology do you use to manage your social connections?
- How much effort do you put into maintaining these connections?
- What may stop you from building social connections?
- What may motivate you to build social connections?
- What social activities do you usually engage in?
- Are you satisfied with the things you do? Why?
- Are there any social activities you would like to start doing, resume doing, or do more of?
- What difficulties do you have in doing these activities? (e.g., memory, language, attention, visuospatial skills, executive/planning)
- How do you cope with your difficulties?
- What kind of technology do you use to manage your social participation?
- How does it help?
- If you woke up tomorrow and did not have any difficulties, what would be different?
- As you mentioned, what do you want to change the most?
- Do you join social groups or clubs?
- Where do the social groups meet?
- How do you evaluate which social group is suitable for you?
- How do you feel when you are in these social groups? Could you describe it?
- Do you have anything further to explain?

**Stage 1 Interview**

- How long have you been a therapist?
- What interventions do you usually provide?
- What instrumental activities of daily life are difficult for people with MCI?
- What social connection circle do you suggest they maintain?
- If you want to enhance the social connections of people with MCI, what would you suggest they do?
- In your opinion, what may stop them from building social connections?
- In your opinion, what may motivate them to build social connections?
- Which social activities are most popular?
- What attributes of social activities attract them?
- What kind of social activities do you suggest they do more of?
- What difficulties do they have in doing these activities? (e.g., memory, language, attention, visuospatial skills, executive/planning)
- What will you suggest they do to cope with these difficulties?
- What instrumental activities of daily life are difficult for people with MCI?
- What will you suggest they change first?
- Which social groups are most popular?
- Where do the social groups meet?
- Which kind of social group do you suggest they join?
- How does participation generate a sense of belonging?
- Do you have anything further to explain?

**Stage 3 Interview**

- Which feature do you like?
- Which feature do you dislike?
- Please give some suggestions for each design.
- What should be improved?
- Have you encountered any difficulties? Can you show me and explain?
